# Decoupling the Good from the Bad: Translational Strategies for Strigolactone Application in Agriculture

**DOI:** 10.3390/biology15151263

**Published:** 2026-08-01

**Authors:** Yanting Wang, Yanni Zhao, Ranran Liu, Shulei Wang

**Affiliations:** 1 College of Agriculture and Biology, Liaocheng University, Liaocheng 252000, China; liuranran0826@163.com (R.L.); wangshulei@lcu.edu.cn (S.W.); 2 Yangling Demonstration Zone Bureau of Modern Agriculture and Rural Revitalization, Yangling 712100, China; zhaoyanni918@163.com

**Keywords:** strigolactone, decoupling, parasitic weed control, abiotic stress tolerance, plant architecture

## Abstract

Strigolactones (SLs) are multifunctional plant metabolites that govern shoot architecture, facilitate symbiosis with arbuscular mycorrhizal fungi, and trigger seed germination of parasitic weeds, making them attractive targets for crop improvement. Their agricultural potential has been validated in field trials for parasitic weed suppression, drought resilience, and grain yield improvement. However, a major challenge is decoupling their beneficial effects from undesirable functions. To address this, we adopt a precision intervention framework distinguishing two strategies: functional decoupling, which separates beneficial from detrimental SL activities; and situational decoupling, which exploits detrimental functions in controlled contexts. We evaluate progress across parasitic weed control, abiotic stress mitigation, and agronomic trait optimization. We also identify scientific gaps and practical barriers limiting translation and critically assess emerging solutions to these barriers. By critically analyzing where decoupling works and what trade-offs limit its success, this review aims to guide sustainable implementation of SL-based technologies in agriculture.

## Simple Summary

Plants produce natural compounds called strigolactones (SLs) that have both helpful and harmful effects. On one hand, these compounds help plants survive droughts and form beneficial partnerships with soil fungi that provide nutrients. On the other hand, they attract parasitic weeds that steal water and nutrients from crops, causing billions of dollars in agricultural losses worldwide. This review examines how scientists are working to separate these good and bad effects—a process called decoupling—so that farmers can use SLs to protect their crops without accidentally encouraging weed problems. Research has already achieved success in controlling parasitic weeds through specially designed chemicals that trick weed seeds into germinating without any crops present, as well as through genetically modified crops that produce less of the weed-attracting signal. However, applications for improving drought tolerance, nutrient efficiency, and plant architecture are still at early stages. The review also identifies key scientific gaps and practical challenges—such as high production costs and formulation instability—that must be addressed before SL-based technologies can be widely adopted. By analyzing what works and what limits success, this review aims to guide the development of sustainable agricultural solutions that enhance crop productivity while reducing reliance on chemical inputs.

## 1. Introduction

Global agriculture faces the urgent challenge of balancing three core objectives: feeding a growing global population, adapting to climate change-induced extreme weather events, and reducing environmental degradation from high-input farming practices [1]. Abiotic stresses such as drought, nutrient deficiency, and soil salinization continue to cause substantial yield losses, while parasitic weeds (e.g., *Striga* and *Orobanche* spp.) infest over 60 million hectares of farmland worldwide, causing billions of dollars in economic damage and threatening food stability, particularly in sub-Saharan Africa and Asia [2].

Strigolactones (SLs) are a textbook example of a “friend and foe” molecule. As foes, they were first identified as germination stimulants for the parasitic weed *Striga lutea* following the isolation of strigol and strigyl acetate from the root exudate of cotton, a non-host plant for *Striga* [3]. Subsequently, various additional SLs were detected in several host plants including sorghum (*Sorghum bicolor*), maize (*Zea mays*), and proso millet (*Panicum miliaceum*), and these SLs trigger seed germination of *Striga*, *Phelipanche*, and *Orobanche* species [4,5,6,7]. These findings established the key role of SLs in host–parasite interactions but raised an intriguing question: what selective pressures drive host plants to produce and release compounds that attract their own parasites?

As friends, a selective advantage of SL production was identified in 2005: 5-deoxystrigol (5DS) isolated from *Lotus japonicus* root exudates acts as a branching factor for arbuscular mycorrhizal fungi (AMF), promoting root colonization [8]. These essential symbiotic fungi supply inorganic nutrients to more than 80% of land plant species and also enhance plant resistance to various stresses, including drought and pathogen attack [9,10]. Recent studies have further elucidated the mechanistic basis of AMF-mediated stress protection: *Funneliformis mosseae* enhances drought tolerance in maize through genotype-specific transcriptional reprogramming [11], while *Septoglomus constrictum* colonization mitigates heat stress in tomato (*Solanum lycopersicum*) by reducing oxidative damage and modulating stress-responsive gene expression [12]. Nevertheless, the fact that non-mycorrhizal plants like *Arabidopsis thaliana* also produce SLs pointed to additional functions beyond mycorrhizal symbiosis [13]. The evolutionary paradox began to be resolved when a new hormonal role for SLs was uncovered in 2008: SLs were identified as a previously unknown graft-transmissible signal that inhibits shoot branching [14,15]. Since then, additional hormonal functions of SLs have been identified, including regulating root architecture [16], promoting secondary growth [17], enhancing leaf senescence [18], and regulating responses to abiotic and biotic stress [19]. This functional duality, simultaneously mediating plant–environment interactions and endogenous developmental programs, positions SLs as promising targets for agricultural intervention.

Despite these advances, the translation of SL-based technologies from laboratory to field has been uneven. The most advanced applications are in parasitic weed control, where field trials have validated the efficacy of formulated SL analogs in reducing *Striga* emergence in maize, pearl millet and sorghum fields [20,21], and where transporter-edited crops have demonstrated resistance to *Striga* and *Orobanche* without compromising yield [22,23]. By contrast, applications for abiotic stress mitigation remain largely confined to greenhouse or pot experiments, with field-scale validation still scarce [24]. To date, no SL-based product has been commercially registered, reflecting a persistent translational gap attributable to high production costs, formulation instability, off-target effects, and limited long-term ecological data [25,26].

A key functional obstacle underlying this translational gap is that SLs are multifunctional: a single compound or pathway can simultaneously trigger beneficial and detrimental outcomes. This functional promiscuity has been a major obstacle to agricultural deployment—for example, applying GR24 to enhance drought tolerance may simultaneously suppress tillering, reducing yield potential. Therefore, decoupling—the separation of beneficial functions (stress tolerance, symbiosis, yield optimization) from detrimental ones (parasitic weed attraction, excessive branching inhibition)—has emerged as a central research priority to overcome this limitation. Here, we extend the discussion to encompass a broader framework of precision intervention in SL functions for agricultural benefit, within which decoupling represents one key category. Specifically, we distinguish two complementary strategies: (i) functional decoupling, which separates beneficial from detrimental SL activities through chemical and genetic approaches; and (ii) situational decoupling, which exploits detrimental SL functions in a controlled context to achieve a beneficial outcome. We evaluate progress across parasitic weed control, abiotic stress mitigation, and agronomic trait optimization, highlighting where these precision intervention strategies have been achieved at the field level and where applications remain at the proof-of-concept stage. We also identify practical barriers to commercialization and fundamental scientific gaps that continue to hinder targeted decoupling, and critically assess emerging solutions to these barriers. Unlike previous reviews that focus primarily on molecular mechanisms [19,27], we critically analyze where decoupling works and what trade-offs limit its success, with the goal of guiding future efforts towards practical implementation of SL-based technologies in sustainable agriculture.

## 2. Molecular Basis for Functional Decoupling: Where Can We Intervene?

### 2.1. Structural Diversity: Canonical Versus Non-Canonical SLs—A Natural Functional Separation

Plants produce and exude unique mixtures of SLs, with over 35 distinct SLs identified across diverse plant species to date [28]. Structurally, SLs are broadly classified into two categories: canonical SLs contain a tricyclic lactone (ABC-ring) linked to a butenolide (D-ring) via an enol ether bridge, whereas non-canonical SLs lack one or more of the A, B, or C rings but retain the enol ether-D ring moiety (Table 1) [29,30,31,32].

Crucially, these structural classes correspond to distinct biological functions. Canonical SLs predominantly act as rhizospheric signals, inducing parasitic weed germination and promoting AMF hyphal branching (Table 1) [33]. By contrast, non-canonical SLs are sufficient to regulate shoot branching as endogenous phytohormones (Table 1) [33,34]. This functional separation is supported by multiple lines of evidence. The rice (*Oryza sativa*) *os900* mutant, which accumulates only non-canonical SLs, exhibits normal shoot branching but shows significantly reduced *Striga* seed germination activity [33]. Similarly, the tomato *cyp722c* mutant, lacking the canonical SL orobanchol, displays no high-branching or dwarf phenotype [35]. Furthermore, chemical inhibition of canonical SL biosynthesis using TIS108 at 10 μM effectively reduces *Striga* infestation without adversely affecting rice growth, although dose-dependent effects should be considered at higher concentrations [33]. These findings collectively indicate that canonical SLs are not the major branching/tillering inhibitory hormones [34]. Moreover, because non-canonical SLs are evolutionarily ancient and conserved from bryophytes to angiosperms, whereas canonical SLs emerged later and are restricted to certain angiosperms, it is plausible that non-canonical SLs represent the ancestral hormonal signals for developmental regulation [34].

### 2.2. Biosynthetic Nodes as Editing Targets

**Figure 1:**
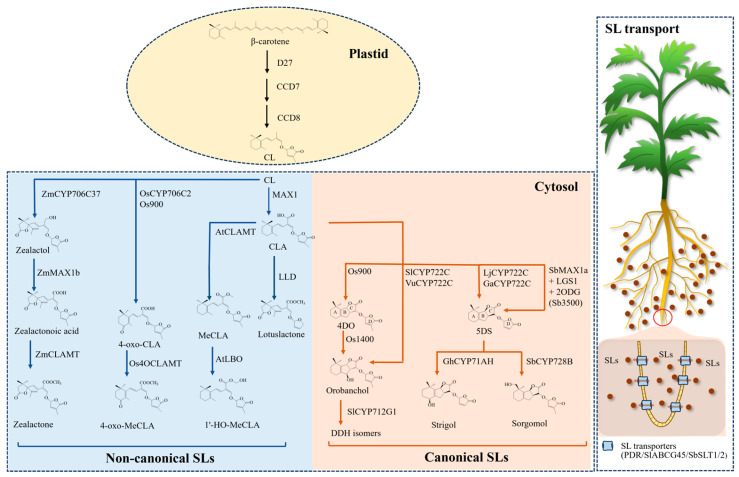
Species-specific strigolactone biosynthetic pathways and rhizosphere transport as intervention targets for functional decoupling. The **upper** panel illustrates the core biosynthetic pathway (black arrows): β-carotene is converted to carlactone (CL) via DWARF27 (D27), CAROTENOID CLEAVAGE DIOXYGENASE 7 (CCD7) and CAROTENOID CLEAVAGE DIOXYGENASE8 (CCD8) in plastids. CL is exported to the cytosol and converted to carlactonoic acid (CLA) by MAX1 (CYP711A). Downstream branches mediated by species-specific enzymes lead to canonical SLs (pink background) and non-canonical SLs (blue background). Canonical SLs (orobanchol, strigol, 5-deoxystrigol (5DS), 4-deoxyorobanchol (4DO), sorgomol) act as rhizosphere signals, inducing parasitic weed seed germination and promoting arbuscular mycorrhizal fungal (AMF) hyphal branching. Non-canonical SLs (methyl carlactonoate (MeCLA), 1′-HO-MeCLA, 4-oxo-methyl carlactonoate (4-oxo-MeCLA), and lotuslactone) primarily regulate shoot branching/tillering, though some also exhibit weak rhizosphere activity (zealactone, zealactol, zealactonoic acid). The **right** panel depicts SLs (the brown dots) secretion into the rhizosphere, mediated by ABCG transporters, including PDR1 (*Petunia hybrid*), SlABCG45 (tomato), and SbSLT1/2 (sorghum). DDH, didehydro-orobanchol. The pathway from CL to canonical and non-canoncial SLs is indicated by blue and orange arrows, respectively.

The core SL biosynthetic pathway is conserved across land plants: β-carotene is converted to carlactone (CL) via β-CAROTENE ISOMERASE (D27), CAROTENOID CLEAVAGE DIOXYGENASE 7 (CCD7), and CCD8, and CL is then exported to the cytosol, where cytochrome P450 (CYP450) and other enzymes generate structural diversity (Figure 1) [36,37].

Key enzyme families provide tractable editing targets for reshaping SL profiles. CYP711A, also known as MORE AXILLARY GROWTH 1 (MAX1), catalyzes the conversion of CL to carlactonoic acid (CLA) (Figure 1) [38]. In rice, specific MAX1 homologs—Os900 and Os1400—drive the production of canonical SLs 4-deoxyorobanchol (4DO) and orobanchol (Figure 1) [38]. Notably, the rice *os900* mutant lacks canonical SLs but maintains normal tillering, demonstrating that selective inactivation can alter SL profiles without affecting plant architecture [33]. CYP722C directly converts CLA into canonical SLs in tomato, cowpea *(Vigna unguiculata*), cotton, and *L*. *japonicus*, making it an attractive editing target to reduce parasitic weed germination without disturbing tillering control (Figure 1) [35,39,40]. Species-specific P450s such as maize CYP706C37 and rice OsCYP706C2, together with MAX1 and CLA METHYLTRANSFERASE (CLAMT), generate non-canonical SLs, which may affect traits like parasitic plant germination and mycorrhization (Figure 1) [28,41]. In addition, non-CYP450 enzymes—CLAMT and LATERAL BRANCHING OXIDOREDUCTASE (LBO) in *Arabidopsis*, and LOTUSLACTONE DEFECTIVE (LLD) in *L*. *japonicus*—contribute to SL diversification (Figure 1) [40,42]. Collectively, these enzymes provide a suite of molecular targets for precision breeding.

### 2.3. Transporters as New Targets

SL secretion into the rhizosphere is mediated by ABC transporter G (ABCG) transporters. The first identified was PLEIOTROPIC DRUG-RESISTANT 1 (PDR1) in petunia, required for SL secretion and AMF symbiosis [43]. Recent studies have uncovered SL-specific transporters in crop species. In sorghum, SORGHUM SL TRABSPORTER 1 (SbSLT1) and SbSLT2 mediate SL secretion and their knockout reduces *Striga* germination without affecting plant architecture [22]. In tomato, SlABCG45 and SlABCG44 regulate root exudation, shoot transport, and resistance to *Phelipanche* and *Orobanche* [23].

Targeting SL transport offers a more precise decoupling strategy than blocking biosynthesis, as it reduces rhizosphere SL levels and parasitic weed germination while preserving intracellular SL functions essential for plant architecture. However, because AMF symbiosis depends on SL secretion, reduced SL exudation may also compromise mycorrhizal colonization—a trade-off observed in petunia *pdr1* mutants [33]. Partial knockdown or tissue-specific editing could potentially fine-tune this balance.

### 2.4. Signaling Nodes as Regulatory Decoupling Targets

SL perception is mediated by the α/β-hydrolase receptor DWARF14 (D14), which, together with the F-box protein D3/MAX2 and the transcriptional repressor D53/SMXLs, forms a core signaling complex that regulates shoot branching and stress responses [44,45,46,47,48]. Upon SL binding, D53/SMXLs are targeted for degradation, releasing transcription factors such as IPA1 from repression [48].

Several nodes in this pathway are amenable to regulatory decoupling. At the receptor level, nitrogen-induced phosphorylation of D14 modulates its stability, enabling nutrient-specific tillering adjustment without permanently altering plant architecture [49]. At the repressor level, SMXL6/7/8 act as master repressors of shoot branching, whereas SMAX1 and SMXL2 regulate seedling development [50,51]. Notably, both SMAX1 and SMXLs negatively regulate drought tolerance [52,53]. Phosphorylation of SMXL6/7/8 by plant-specific casein kinases (AELs) provides an additional layer of regulation [54]. Built-in signal termination mechanisms—D3 acts as both an accelerator and brake, feedback expression of *SMXL* genes, and SL hydrolysis via carboxylesterases such as AtCXE15—fine-tune signal duration [49,55,56].

Collectively, these signaling components represent promising targets for decoupling stress-protective or architectural benefits from constitutive growth penalties.

Nevertheless, because SLs function within a broader hormonal network involving auxin, ABA, and jasmonic acid, modification of SL genes may have unintended consequences through crosstalk. Strategies to minimize such effects—including tissue-specific editing, partial knockdown, and receptor-selective mimics—are discussed in the following sections, where off-target effects and practical barriers are addressed.

## 3. Validated Decoupling Strategies for Agricultural Application

Parasitic plants of the genera *Striga* and *Orobanche* represent some of the most destructive weeds in global agriculture as outlined in Section 1. The germination of their seeds relies on the host secreted SLs [21,57]. Consequently, the best-investigated field-scale application of SL is the control of parasitic weeds, implemented through three distinct approaches that align with the precision intervention framework introduced in Section 1.

First, functional decoupling via genetic strategies involves targeted modification of SL biosynthesis, signaling, or transport genes in host crops to attenuate SL production or exudation, reducing the germination signal perceived by parasitic weeds while preserving beneficial arbuscular mycorrhizal symbiosis. Second, functional decoupling via chemical strategies employs receptor-specific SL mimics that activate only parasitic weed germination pathways without affecting host plant development. Third, situational decoupling deploys synthetic SL analogs or mimics to induce suicidal germination—the premature germination of parasitic weed seeds in the absence of hosts—thereby depleting the soil seed bank without affecting crop growth.

In addition to these deliberate interventions, natural variation in SL profiles can also be exploited for parasitic weed management without genetic or chemical manipulation. Maize varieties secreting higher amounts of zealactol and zealactonoic acid (e.g., genotype CML52, NC358 and NP2222) show greater resistance to *Striga* than those secreting high zealactone (e.g., genotype CML69) [41]. This natural variation offers a resource for breeding without transgenic modification.

### 3.1. Genetic Functional Decoupling: Targeting Biosynthesis

Crop varieties with reduced or impaired SL exudation can exhibit resilience to parasitic plants. For example, low-SL exudation varieties in rice [58], tomato [59], faba bean (*Vicia faba*) [60] and pea (*Pisum sativum*) [61] have been shown to suppress the germination and parasitism of *Striga*, *Orobanche* or *Phelipanche* species. However, complete loss of SLs through genetic manipulation of the SL biosynthetic pathway often leads to undesirable outcomes, including excessive shoot branching, impaired mycorrhizal symbiosis, and reduced yield. For instance, mutation of *Non-dormant Axillary Bud 1* (*NAB1*), an ortholog of *D17* in sorghum, reduces SL biosynthesis and decreases *Orobanche* germination, but also affects plant architecture and grain yield [62]. Thus, complete biosynthesis knockout is not a viable genetic functional decoupling strategy.

A more refined decoupling approach is to shift the SL profile rather than eliminate SLs entirely. The best-characterized example is the natural *LOW GERMINATION STIMULANT 1* (*LGS1*) allele in sorghum, which shifts the major root-exuded SL from 5DS (highly active on *Striga*) to orobanchol (less active on *Striga*) [63]. This change greatly reduces *Striga* germination without affecting mycorrhization status—a successful genetic functional decoupling that preserves beneficial symbiosis [63]. Although the same mutation may pose a risk by stimulating *Orobanche* seed germination in the soil, sorghum is not a natural host for *Orobanche*. Consequently, any *Orobanche* seeds induced to germinate by orobanchol cannot parasitize sorghum and will die. Therefore, to some extent, the *lgs1* mutation may actually reduce the *Orobanche* seed bank in the soil.

In maize, Li et al. (2023) demonstrated that reducing the activity of ZmCYP706C37, ZmMAX1b and ZmCLAMT1 shifts the SL profile from zealactone towards zealactol and zealactonoic acid, thereby decreasing *Striga* germination and infection [41]. This provides a promising route for breeding *Striga*-resistant maize varieties by reprogramming the endogenous SL blend, representing another example of genetic functional decoupling through modulation of species-specific biosynthetic enzymes.

### 3.2. Genetic Strategies: Targeting Transport

Apart from biosynthesis, the secretion of SLs into the rhizosphere is another factor that influences *Striga* germination and infestation. The petunia *pdr1* mutant exhibited decreased SL secretion from roots and effectively reduced seed germination of root-parasitic weed *Phelipanche ramosa* [43]. Notably, mutation of *PDR1* also reduces AMF colonization in petunia [43], illustrating a trade-off associated with this genetic functional decoupling approach.

While PDR1 plays dual roles in SL secretion and symbiosis, recent breakthroughs in crop species have identified SL transporters that enable more precise genetic functional decoupling. In sorghum, single or double knockout of *SbSLT1* and *SbSLT2* significantly reduced SL content in root exudates, leading to decreased *Striga* germination without adversely affecting plant architecture [22]. In a field plot experiment, both single and double *SbSLT1/2* knockout mutants exhibited significantly reduced *Striga* infestation (67–94%) compared with wild-type plants [22]. Accordingly, both single and double mutants showed lower yield losses and produced significantly higher fresh and straw weights than wild-type controls [22]. These field results demonstrated that *SbSLT1/2* knockout effectively suppresses *Striga* infestation under field conditions, and may thereby mitigate sorghum production losses caused by *Striga* parasitism—representing a successful example of genetic functional decoupling at the transporter level.

In tomato, genetic analysis revealed that *SlABCG45* and its homolog *SlABCG44* are responsible for SL secretion into the rhizosphere, shoot-to-root SL transport, and resistance to *Phelipanche* and *Orobanche* [23]. Knockout of *SlABCG45* in field trials conferred resistance to *Phelipanche* and *Orobanche* species with minor effect on yield, resulting in a 30% yield increase in a *Phelipanche*-infested field [23]. This further confirms transporter editing can achieve genetic functional decoupling by reducing rhizosphere SL signaling while preserving intracellular SL functions essential for plant architecture.

Together, these studies indicate that identifying SL-transporter genes and elucidating their mechanisms in other staple crops (such as wheat (*Triticum aestivum*), maize, and rice) opens new avenues for sustainable control of parasitic plants in agriculture. Comparative genomics and transcriptomics under phosphate deficiency, a condition that strongly induces SL exudation, offer a promising route to discover these transporters and extend genetic functional decoupling strategies to additional crops.

### 3.3. Chemical Functional Decoupling: Receptor-Specific SL Mimics

Receptor-specific SL mimics represent a form of chemical functional decoupling: they exploit receptor differences between host plants and parasitic weeds to activate only target pathways, thereby avoiding effects on host plants and AMF symbiosis.

A prime example is Sphynolactone-7 (SPL7), which was developed to target ShHTL7, one of the most sensitive *Striga* receptors, responding to picomolar concentrations of SLs when heterologously expressed in *Arabidopsis* [64,65]. SPL7 exhibits high affinity for ShHTL7 but very low affinity for the SL receptor D14 in *Arabidopsis* [66]. Consequently, SPL7 application to SL biosynthetic-mutant *max4* did not restore its increased branching phenotype, and SPL7 showed 800-fold lower potency for inducing AMF hyphal branching compared to GR24 [66]. Importantly, soil treatment with SPL7 before planting maize effectively reduced *Striga* emergence [66]. These characteristics make SPL7 an effective tool for chemical functional decoupling, selectively triggering *Striga* seed germination without disrupting host crop growth, AMF symbiosis, or soil microbial communities.

Beyond species-specific mimics like SPL7, recent advances have yielded functionally specialized SL mimics that separate hormonal activity from rhizosphere signaling. For example, 4-Br debranone (4BD) suppresses tillering and shoot branching in SL-deficient mutants of rice and *Arabidopsis* but shows more than 50-fold reduced efficacy in inducing parasitic weed seed germination compared to GR24, indicating host-specific activity [67]. AR36 exhibits hormonal function in inhibiting shoot and root architecture, yet lacks activity in inducing seed germination of *Phelipanche* and *Striga* or hyphal growth of AMF [68]. 113D10 acts as a D14-dependent SL agonist but fails to stimulate parasitic seed germination [69]. These functionally specialized agonists demonstrate that chemical functional decoupling can be tailored for specific agricultural targets—either to optimize plant architecture or to manage parasitic weeds—with minimal off-target effects.

While the strategies described above have achieved notable successes in parasitic weed control, they still have limitations. Trade-offs such as species-specific efficacy and inconsistent field performance have been observed in certain cases. A more systematic discussion of these off-target effects, along with strategies to mitigate them—including receptor-selective mimics and precision gene editing—is provided in Section 5.2.

### 3.4. Situational Decoupling: Suicidal Germination

As introduced above, suicidal germination represents a form of situational decoupling: it exploits SL’s ability to trigger parasitic weed seed germination in a controlled context—the absence of a host—where the detrimental effect is converted into a weed-control benefit without affecting the crop plant. By depleting the soil seed bank, this strategy uses the detrimental function of SLs as a tool for agricultural benefit, rather than separating it from beneficial functions.

The structural complexity and stereochemistry of natural SLs render their large-scale chemical synthesis impractical, and their intrinsic instability hinders feasible extraction from plant sources for agricultural applications. To overcome these limitations, synthetic SL analogs and mimics have been developed. Several of these have been validated in field trials for suicidal germination.

SL analogs, which are structurally similar to natural SLs, are widely used to stimulate the germination of various parasitic weeds, including *Striga* and *Orobanche* species. Representative analogs include GR24, Nijmegen-1, Nijmegen-1 Me, MP1 and MP3. In field trials targeting hemp broomrape (*Orobanche ramosa* L.) in tobacco (*Nicotiana tabacum* L.), application of Nijmegen-1 and Nijmegen-1 Me resulted in a ≥95% reduction in broomrape emergence in four of nine field trials, and a 60–70% reduction in two trials [70]. Similarly, the application of Nijmegen-1, MP3, and MP16 reduced *Striga* emergence in pearl millet and sorghum fields in Burkina Faso, achieving reductions of 43%, 33%, and 41% in pearl millet, and 60%, 52%, and 11% in sorghum, respectively [21].

A recent field study in Kenya evaluated the efficacy of two formulated SL analogs, MP3 and Nijmegen-1, in both liquid (emulsifiable concentrate, EC) and granular (GR) formulations for *Striga* management in maize fields. In laboratory germination bioassays, the EC formulation of MP3 and Nijmegen-1 induced 45–56% *Striga* seed germination, which was approximately 24% higher than the GR formulation (46%) [20]. Under greenhouse pot conditions, the EC formulation of MP3 reduced *Striga* emergence by approximately 75%, about 16% higher than the GR formulation (63%), while Nijmegen-1 showed similar activity with both formulations, achieving a 77% reduction [20]. In farmer-managed field trials across three *Striga*-infested regions in Kenya, the effectiveness varied by location. In Busia, both analogs in GR formulation reduced *Striga* emergence by 45–56%, while EC formulation achieved 32–45% reduction. In Siaya, both formulations of MP3 and Nijmegen-1 showed comparable reductions of 36–40%. In Homa Bay, MP3 in EC formulation achieved up to 65% reduction, whereas Nijmegen-1 showed only 2–17% reduction with both formulations [20]. These results highlight that the efficacy of suicidal germination agents can be influenced by field-specific factors such as *Striga* seed density, soil properties, and local *Striga* ecotypes.

### 3.5. Comparative Summary of Precision Intervention Strategies

The four strategies discussed above, namely biosynthesis editing, transporter editing, receptor-specific mimics, and suicidal germination, differ substantially in their mechanism, applicability, and stage of field validation. They can be grouped according to the precision intervention framework introduced in the introduction section: functional decoupling (biosynthesis editing, transporter editing, and receptor-specific mimics) aims to separate beneficial from detrimental SL activities; situational decoupling (suicidal germination) exploits detrimental SL functions in a controlled context to achieve a beneficial outcome.

**Figure 2:**
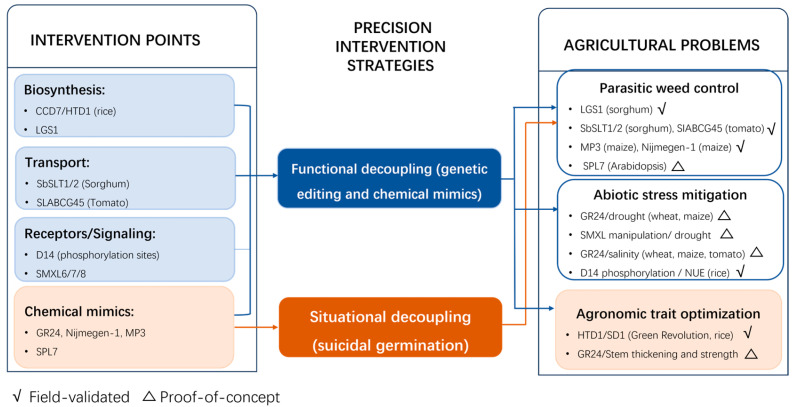
Decision flow chart for decoupling strategies in SL agricultural applications. The chart illustrates the logical connections between molecular intervention points, translational strategies, and agricultural problem domains. The **left** panel lists the key intervention nodes across four categories—biosynthesis, transport, receptors/signaling, and chemical mimics. The **middle** panel organizes these nodes into two precision intervention strategies (functional decoupling and situational decoupling). The **right** panel maps the resulting applications to three agricultural problem domains: parasitic weed control, abiotic stress mitigation, and agronomic trait optimization. Arrows indicate the pathways from specific intervention points to their corresponding strategies and applications. Light blue boxes represent genetic intervention targets; light orange boxes indicate chemical mimic targets. Dark blue and dark orange rounded boxes refer to functional decoupling and situational decoupling strategies, respectively. Tick indicates field-validated applications; triangle indicates proof-of-concept or emerging applications.

Table 2 summarizes these strategies to guide selection based on crop, target parasitic weeds, and agricultural context. Each strategy has its own niche. Biosynthesis editing is the most suitable for staple crops where durable, heritable resistance is desired, provided that the edited allele does not compromise yield. Transporter editing currently represents the most promising approach because it uncouples parasitic weed attraction from plant development, as demonstrated in both sorghum and tomato [22,23]. Receptor-specific mimics offer pathway-specific activation with minimal off-target effects. SPL7 has demonstrated efficacy in pot and laboratory experiments as a *Striga*-specific suicidal germination agent, although field-scale validation has not yet been reported. Other mimics (4BD, AR36, 113D10) remain at the laboratory stage. Suicidal germination offers a flexible, non-transgenic option for immediate use, particularly in regions where genetically modified crops face regulatory hurdles. However, its cost and inconsistent field performance remain challenges. To provide a holistic overview of how the individual intervention points connect to these strategies and agricultural problems, a decision flow chart is presented in Figure 2.

## 4. Underexplored Potential and Fundamental Scientific Gaps

### 4.1. Abiotic Stress Tolerance: Untapped Field Potential and Decoupling Bottlenecks

In contrast to the control of parasitic weeds, which has seen both functional decoupling and situational decoupling validated in field conditions, the application of SLs for alleviating abiotic stress is predominantly at the proof-of-concept level. The key challenge is not simply to enhance stress tolerance, but to achieve functional decoupling—separating the stress-protective effects of SL signaling from its constitutive activation, which would suppress tillering and compromise yield. This section examines the existing evidence for SL-mediated stress protection, outlines instances where functional decoupling has been achieved at the mechanistic level, and emphasizes the translational gaps that must be addressed for effective field deployment.

#### 4.1.1. Drought Stress: Current Evidence and Translational Gaps

SLs have been shown to enhance drought tolerance by promoting stomatal closure and activating antioxidant defenses [71,72]. Exogenous application of GR24 has demonstrated efficacy in multiple crop species, including wheat [73,74,75,76,77], maize [24,78,79], soybean [80], cotton [81], and grapevine [82]. These studies collectively demonstrate that GR24 treatment improves photosynthetic efficiency, reduces oxidative damage, and enhances osmotic balance under water-limited conditions. Notably, the SL analog AB01 has been shown to increase grain yield in field-grown maize under drought [24], representing one of the few field-validated examples. Table 3 summarizes representative studies across crop species and experimental settings.

Many of the studies on exogenous applications face a common issue: GR24 is used as a constitutive treatment, which means that SL signaling is activated regardless of stress conditions. In non-stress situations, this activation may suppress tillering, reduce biomass, and ultimately negate any benefits derived from stress. Rarely have these studies evaluated whether the observed drought tolerance can be achieved without constant suppression of branching, highlighting a significant gap in applying exogenous SL treatments in practical field settings.

Beyond exogenous application, the genetic manipulation of SL pathway components has been investigated as a strategy for improving drought tolerance. At the biosynthesis level, silencing *GhMAX3* and *GhMAX4b* in cotton resulted in impaired drought tolerance, whereas their overexpression was able to rescue the drought-sensitive phenotype [81]. At the receptor level, the ectopic expression of *ZmD14* in *Arabidopsis* led to enhanced drought tolerance [83]. At the repressor level, manipulating SMXL family members has provided valuable insights into the potential benefits and limitations of pathway-specific interventions. The *Arabidopsis smxl6,7,8* triple mutant demonstrated reduced cuticle permeability and decreased water loss under drought stress [84]. However, SMXL6/7/8 are transcriptional repressors that degrade upon SL perception, which releases downstream signaling [50,51]. Their loss-of-function leads to constitutive SL signaling and consequently reduced branching—a trait generally associated with yield penalties in most crops [50,51,85]. Thus, while this mutant provides proof-of-concept for enhancing stress resistance through manipulation of downstream signaling components, its agricultural applicability as a decoupling strategy remains limited unless tissue-specific or conditional manipulation can be achieved. In contrast, disruption of *SMAX1* and *SMXL2* enhanced drought resistance in *Arabidopsis* without apparent effects on shoot architecture [53], suggesting that certain SMXL family members may offer more promising decoupling targets. In cotton, silencing *GhSMXL7* improved resistance to verticillium wilt, hinting at potential applications for drought tolerance as well [86]. However, whether this improvement comes at the cost of growth penalties requires further investigation. At the transcriptional level, overexpression of *MdIAA24* in apple upregulated SL biosynthetic genes and enhanced drought resistance [87]. Meanwhile, the cotton transcription factor GhRAP2.4 activated the SL receptor gene *GhD14*, and its overexpression improved drought tolerance [88].

Beyond individual gene manipulation, multidimensional omics approaches—integrating transcriptomics, metabolomics, and phenotyping—provide a powerful strategy for deciphering the molecular basis of SL-mediated stress responses. These approaches could help identify key regulatory nodes that differentiate stress-protective SL outputs from growth-suppressive ones, thereby guiding more precise decoupling strategies.

#### 4.1.2. Nutrient Deficiency: Regulatory Nodes for Decoupling

Phosphorus (P) and nitrogen (N) are essential for plant growth. SL signaling plays a key role in helping plants adapt to deficiencies in these nutrients. Unlike the continuous GR24 treatments previously discussed in relation to drought, recent studies have pinpointed specific signaling nodes within the SL pathway that can be effectively decoupled for regulatory purposes.

Under N deficiency, phosphorylation of the SL receptor D14 stabilizes the protein, which delays signal termination and restrains tiller bud outgrowth to optimize N allocation [49]. Importantly, modifying D14 phosphorylation sites can maintain or increase tiller numbers while reducing N fertilizer requirements [49]. This exemplifies regulatory decoupling, where nutrient-specific adaptation is achieved without permanently altering plant architecture, providing a promising strategy for breeding nitrogen-use efficient (NUE) varieties.

Under P deficiency, SL signaling downregulates N transporter genes (*OsNRTs*) while upregulating P transporter (*OsPTs*), thereby rebalancing the allocation of N and P resources [89]. This regulatory crosstalk indicates that SL signaling could potentially be manipulated to optimize nutrient balance during deficiency conditions; however, it remains to be determined whether this crosstalk can be exploited without affecting SL-dependent developmental programs.

SLs also influence the assembly of root microbiota in response to P availability. In *Arabidopsis*, SL biosynthesis (*max3-11*) and perception (*d14-1*) exhibited altered root-associated microbial communities under low P conditions, with SLs attracting beneficial *Acinetobacter* spp. [90]. Notably, an isolated *Acinetobacter* soli strain (CP-2) from these communities enhanced plant growth in cocultivation experiments [90]. These findings underscore the potential for developing SL-based biostimulants or microbial inoculants to enhance nutrient-use efficiency in sustainable agriculture. However, since these mutants are globally defective in SL biosynthesis or perception globally, it remains an unresolved question whether the observed changes in microbial community can be dissociated from SL-mediated developmental effects.

Characterizing the role of SLs in plant–microbe interactions at a systems level will require integrated omics approaches. By combining root exudate metabolomics with plant and fungal transcriptomics, researchers can identify the key metabolites and signaling pathways that govern AMF symbiosis in a species-specific manner. Such studies would clarify whether the SL-dependent recruitment of beneficial microbes can be separated from SL-mediated developmental effects, which is a critical question for the development of biostimulants.

#### 4.1.3. Salinity Stress: GR24 Efficacy and the Same Decoupling Bottleneck

Soil salinization poses a significant challenge to crop productivity globally. The application of exogenous GR24 has been shown to mitigate salt-induced damage across various crop species. In maize, treating seeds with GR24 markedly enhanced root length and overall leaf area when subjected to salt stress [91]. In wheat, foliar spraying of GR24, either alone or in combination with the plant growth-promoting rhizobacterium (PGPR) *Bacillus velezensis* UTB96, resulted in reduced Na^+^ accumulation, increased K^+^ levels, and improved growth and yield traits under saline conditions [92]. For tomato, foliar application of GR24 significantly boosted seedling growth and augmented endogenous trehalose and starch levels during salt stress; importantly, the co-application of the SL biosynthesis inhibitor TIS108 negated these beneficial effects, indicating that SLs are essential for salt tolerance [93]. In rapeseed (*Brassica napus* L.), GR24 treatment partially alleviated the reductions in shoot and root growth as well as photosynthetic parameters caused by salinity [94]. In apple rootstock *Malus hupehensis*, root drench treatment with GR24 effectively mitigated salinity–alkalinity stress, as demonstrated by increased chlorophyll content and photosynthetic rate [95]. This suggests that GR24 application can enhance abiotic stress tolerance in fruit trees, although further field validation is required.

However, similar to drought stress, these studies involving exogenous applications face a decoupling bottleneck: GR24 is used as a constitutive treatment that activates SL signaling irrespective of stress status. The potential for achieving salt tolerance without the constitutive suppression of shoot branching—especially in field conditions—remains largely untested. Additionally, the majority of these studies are limited to controlled greenhouse or pot experiments, highlighting the urgent need for validation at the field-scale.

In addition to exogenous applications, genetic manipulation of SL signaling components has been investigated as a means of improving salt tolerance. For instance, overexpressing the soybean SL signaling gene *GmMAX2a* in *Arabidopsis* enhanced root growth, biomass, and the expression of various stress-responsive genes under both salt and alkaline conditions [96]. However, since *MAX2* is a central component of SL signaling, its overexpression may trigger global SL responses, potentially leading to growth penalties. Whether this approach can yield stress-specific effects without compromising plant architecture remains to be determined.

### 4.2. Agronomic Trait Optimization: Historical Lessons and Underexploited Opportunities

Plant shoot architecture, which includes factors such as branching/tillering, plant height, and stem strength, directly influences crop yield and management [85]. SLs play a crucial role in regulating these traits, and specific genetic variations in SL pathways have been utilized in breeding programs [24,97]. However, unlike certain strategies for controlling parasitic weeds, where decoupling has been intentionally engineered (e.g., SPL7 mimics or transporter editing), the optimization of agronomic traits through SL modulation has largely resulted from unintentional selection or remains at the proof-of-concept stage.

During the Green Revolution, the partial loss-of-function allele *HTD1* (*CCD7*), which enhances tiller number without compromising seed set, was co-selected alongside the gibberellin-related semi-dwarf allele *Semi-dwarf 1* (*SD1*) to create high-tillering, semi-dwarf rice varieties [98]. This serves as a classic example of how modulating SL biosynthesis can improve plant architecture for greater productivity. However, this instance represents unintentional decoupling: the *HTD1* allele was selected for its positive effect on tillering, yet the underlying mechanism was not understood at the time, and the trade-offs with other SL-mediated functions were not systematically assessed.

Beyond tillering, SLs also promote cambium activity, which is crucial for stem thickening and strength [24]. In *Arabidopsis*, SL-deficient mutants (*max1*, *max3*, and *max4*) exhibit reduced cambium activity, while local application of GR24 stimulates divisions in cambial cells [17]. This stimulatory effect is observed in woody species as well; for instance, treatment with GR24 in *Eucalyptus globulus* led to an increase in the production of secondary vascular tissues [17]. Additionally, in cherry rootstocks, SL treatment resulted in enhanced stem diameter and strength—traits that are vital for mechanized harvesting and resistance to lodging [99].

These findings indicate that SLs could be utilized to enhance stem strength in both fruit and forest trees, as well as improve lodging resistance in cereal crops. However, the specific SL signals that regulate cambium activity remain unclear, and there is still a lack of field-scale validation in major cereal crops. This underscores the necessity for further research to explore the potential of SL-mediated stem strengthening.

Looking ahead, understanding the regulatory mechanisms behind SL-controlled development will necessitate systems-level approaches. Multi-omics methodologies can reveal how SLs coordinate the regulation of shoot branching and root architecture by integrating signals from other phytohormones, such as auxin and cytokinin. This knowledge would support the development of targeted interventions that can alter specific architectural traits while minimizing pleiotropic effects.

### 4.3. Fundamental Scientific Gaps

As discussed in Section 2.1, canonical and non-canonical SLs demonstrate a natural functional separation, with the former primarily acting as rhizospheric signals and the latter being sufficient to regulate shoot branching. However, several fundamental questions regarding SL diversity remain unanswered.

First, the SLs identified to date have predominantly been characterized from root exudates, revealing remarkable structural diversity across and within plant species. Yet the chemical identity of the SL that functions as an endogenous hormone in above-ground tissues remains elusive.

Second, the biological significance of producing multiple SLs within a single plant species is not well understood. Most non-parasitic plants possess only a single SL receptor (D14), raising the question of whether different SLs can induce distinct receptor conformations and thereby elicit pathway-specific responses. If different SLs cause varying conformational changes in D14—a scenario reminiscent of signal transduction in animal systems—this would have profound implications for agriculture, potentially allowing for the modulation of specific SL responses without affecting others. Alternatively, some SLs may merely be biosynthetic intermediates or byproducts rather than functionally significant signaling molecules, a possibility that has been suggested but remains experimentally untested in the context of SL diversity.

Third, although more than 35 SLs have been identified to date, biosynthetic pathways have been fully elucidated for only a small subset, including CL, CLA, 4DO, orobanchol, DDH, 5DS, strigol, sorgomol, zealactone, MeCLA, and lotuslactone. The downstream diversification steps that lead to the generation of some canonical and most non-canonical SLs remain largely unknown. Even for those SLs with established pathways—such as zealactone and zealactol/zealactonoic acid in maize—the regulatory mechanisms controlling metabolic flux between different branches are still poorly understood [41]. Elucidating these pathways would deepen our understanding of the functional significance of SL structural diversity and facilitate the rational design of SL-based agrochemicals for tailored agricultural applications.

While a few successful cases, such as SL profile-modified crops that confer resistance to parasitic weeds, have demonstrated the feasibility of selective SL manipulation, the broader potential of leveraging SL specificity remains largely untapped. Realizing this potential will require a more comprehensive understanding of SL diversity.

Beyond the chemical diversity of SLs, another layer of complexity involves how these signals are recognized by different organisms. Critical gaps in our understanding of SL perception in parasitic weeds and AMF currently impede efforts to separate SL’s beneficial symbiotic function from its detrimental role in germination of parasitic seed.

The native SL receptor(s) in AMF remain completely unidentified, and the ligand selectivity among diverse AM fungal taxa is uncharacterized. This lack of understanding hinders the development of AMF-specific SL mimetics that would avoid activating HTL/KAI2d receptors in parasitic weed. To address this gap, we propose that affinity-based receptor pull-down techniques, utilizing biotinylated or photoaffinity-labeled SL probes in conjunction with LC-MS/MS proteomics, represent one of the most promising experimental strategies for identifying SL-binding proteins from AM fungal spores or hyphae.

Moreover, the functional diversification of expanded HTL/KAI2d paralogs in *Orobanchaceae* parasites, along with their downstream germination-specific signaling pathways, is still poorly understood. This limits the potential to create parasite-selective SL antagonists that do not interfere with the endogenous SL signaling in crops or disrupt AM symbiosis.

Lastly, the molecular mechanisms underlying the differential perception of SLs among various parasitic genera are not well defined. For example, it remains unclear which structural features of SLs dictate selectivity between *Striga* versus *Orobanche* receptors. Therefore, comparative studies examining receptor structure and function across these genera are urgently needed to inform the design of genus-selective germination stimulants.

Among the gaps outlined above, we consider three to be of the highest priority for accelerating agricultural application. First, elucidating the biosynthetic pathways of non-canonical SLs would enable marker-assisted breeding for *Striga* resistance without the need for transgenic modification. Second, understanding the structural basis of receptor selectivity across parasitic genera would facilitate rational design of genus-specific germination stimulants. This addresses the current limitation where a strategy effective against *Striga* may not work against *Orobanche*. Third, identifying the SL receptor in AM fungi would pave the way for designing SL analogs that promote symbiosis without triggering the germination of parasitic plant seed—a significant breakthrough in decoupling these processes. While all gaps are scientifically important, these three offer the most direct path to solutions that can be implemented in the field and should therefore receive focused research attention.

Collectively, these knowledge gaps—encompassing both the structural diversity and the perception of SLs by various organisms—create a tripartite trade-off among suppressing parasitic weeds, promoting AMF, and preserving the endogenous functions of SL hormones. This represents a core bottleneck that must be overcome for the successful agricultural deployment of SL-based technologies.

## 5. Future Prospects and Practical Barriers

Intervening in SL biosynthesis, transport and signaling through genetic improvement or chemical application offers significant potential for addressing key agricultural challenges, such as enhancing crop productivity, optimizing nutrient acquisition, suppressing parasitic weeds, and increasing stress resilience. However, various challenges hinder translation into practical applications, including high production costs, formulation instability, off-target effects, and potential ecological impacts. This section reviews these practical barriers and discusses emerging solutions, focusing on their current stage of development—from basic research to near market readiness.

### 5.1. Production and Formulation: From High Cost to Microbial Cell Factories

Despite field trials demonstrating the potential of SLs as agrochemicals, commercial products have not yet reached the market. The primary bottleneck is the absence of a cost-effective source for natural SLs or their synthetic analogs, which further restricts research into their practical applications.

The organic synthesis of natural SLs is complicated due to their intricate structure and stereochemistry, making them commercially unavailable or prohibitively expensive [100]. Even synthetic SL analogs, while less complex, remain costly for widespread commercial adoption [101]. A promising alternative is the development of microbial cell factories through synthetic biology and metabolic engineering. Recent advancements in understanding the SL biosynthetic pathway have facilitated the reconstruction of SL production in heterologous hosts. For instance, a microbial consortium expressing the core SL biosynthetic pathway in *Escherichia coli* and *Saccharomyces cerevisiae* achieved a 125-fold increase in canonical SL titers [102]. Further engineering of such platforms could enable cost-effective and scalable production of natural SLs. However, significant economic challenges persist: current titers are still well below industrial viability (typically >1 g/L for commercial bioproduction), and downstream purification costs from complex fermentation broths remain a major cost driver [103]. Additionally, the high cost of fermentation infrastructure and the necessity for strain stability across multiple production cycles present further barriers to large-scale manufacturing [103]. Nevertheless, as production costs for structurally simpler SL analogs have decreased by over 90% through optimized synthetic routes [24], similar cost reductions may be attainable for microbial platforms with continued investment in strain engineering and bioprocess development. At present, this approach remains in the research and early development phase, with commercial viability yet to be established.

Beyond production, the poor water solubility of SL analogs, attributed to their low polarity, limits their application in large-scale agricultural use as agrochemicals [104]. Nanoencapsulation or slow-release systems provide a strategy to enhance stability and bioavailability. For instance, encapsulating the phthalimide–lactone (PL) SL mimic in polyvinyl alcohol (PVA) nanoparticles resulted in a 27-fold improvement in water solubility compared to pure PL while preserving biological activity [105].

Despite this promise, the economic viability of nanoformulations hinges on several factors: (i) the cost of nanocarrier materials (such as biodegradable polymers and lipids) must be weighed against potential savings from reduced active ingredient usage; (ii) large-scale manufacturing of uniform nanoparticles necessitates specialized equipment and stringent quality control, currently leading to significant costs compared to conventional formulations [106,107]; and (iii) the regulatory approval pathways for nanopesticides and nano-biostimulants are still evolving, introducing uncertainty into commercialization timelines [108]. Recent estimates indicate that nanoparticle encapsulation could reduce active ingredient usage by up to 30% while maintaining efficacy [109], presenting potential cost savings that might counterbalance higher production costs. However, these advantages require validation under field conditions across various crops and environments. This formulation technology is still in the early development stage, necessitating further optimization and field validation before commercial deployment.

### 5.2. Off-Target Effects: A Major Challenge for SL Analog Application

SLs serve dual roles as both endogenous plant hormones that regulate development and as rhizosphere signaling molecules in the rhizosphere that mediate interactions with symbiotic and parasitic organisms. The application of SL analogs may lead to unintended consequences. While these compounds can enhance stress resilience in plants, they may also disrupt normal growth and result in reduced yield.

To address these potential drawbacks, two complementary strategies have emerged. Chemically, researchers are focusing on receptor specificity to design targeted SL analogs that minimize off-target activity. Functionally specialized mimics like SPL7, 4BD, AR36, and 113D10 illustrate how precise chemical design can facilitate pathway-specific activation (see Section 3.1). Among these, SPL7 has progressed to field-validated proof of concept, while the others remain in the laboratory phase. On the genetic front, precision editing provides a means to reduce off-target effects in host plants. A more nuanced approach involves the partial reduction in transporter expression through promoter editing or the introduction of weak alleles to fine-tune SL levels, which may help deter parasitic weeds while preserving AMF symbiosis. Transporter editing has already been validated in field trials with sorghum and tomato, positioning it as the closest genetic strategy to market readiness. Additionally, stacking transporter edits with other resistance genes could offer durable, broad-spectrum protection, representing a promising avenue for future crop improvement, although it still remains within the research phase.

### 5.3. Ecological Risks and Regulatory Hurdles for SL Agrochemicals

SLs are biodegradable, and their metabolites may accumulate in soil or aquatic systems. Currently, toxicity assessments of SLs concerning aquatic life and terrestrial organisms are limited, and their environmental fate—such as degradation half-life and soil mobility remains poorly understood [25]. Many countries require extensive safety and efficacy data for registration of agrochemicals. The lack of long-term toxicity and environmental impact studies for SLs has hindered their official approval for commercial use in major agricultural markets, underscoring the urgent need for comprehensive ecotoxicological evaluation before widespread field deployment. This area remains at an early stage, with foundational data being generated.

### 5.4. SL–Microbiome Interactions as a Biostimulant Platform

Looking beyond current applications, SL-based technologies have the potential to evolve into sustainable, eco-friendly biostimulants that are integrated with microbial partners. Understanding how SLs influence root microbiota assembly paves the way for the development of SL-based prebiotics or co-formulations with specific microbial inoculants. For instance, SLs affect root microbiota assembly in response to phosphorus availability, promoting beneficial taxa such as *Acinetobacter* spp. [90]. Such biostimulants could enhance nutrient uptake and stress resilience in field conditions, making this area ripe for long-term research and development. A particularly promising avenue is the combination of SL analogs with AMF or PGPR. Recent studies indicate that the combined application of PGPR, melatonin, and GR24 effectively mitigated carbendazim-induced stress in chickpea seedlings [110]. In wheat, the simultaneous application of GR24 as a foliar spray and PGPR inoculation synergistically alleviated salt stress, improving growth and yield traits beyond what either treatment could achieve alone [92]. In aerobic rice, seed priming with GR24, in conjunction with AMF inoculation, significantly enhanced plant growth and phosphorus uptake under P-deficient conditions [111]. These findings support the notion that integrating SL analogs with beneficial microorganisms represents a promising strategy for developing next-generation biostimulants. Currently, this concept remains in the basic research phase, with an incomplete mechanistic understanding and a lack of field validation.

## 6. Conclusions

In summary, the agricultural utility of SLs is now supported by an expanding body of evidence, both as active agrochemical ingredients and as targets for genetic improvement, with biosynthetic, signaling, and transport mutants all contributing valuable agronomic traits. Field successes in parasitic weed suppression—achieved through suicidal germination agents and transporter-edited crops—have confirmed that decoupling SLs’ beneficial effects from their harmful ones is a practical reality. However, for abiotic stress mitigation and agronomic trait optimization, SL-based applications remain largely at the proof-of-concept stage. Our growing understanding of SL biology has already proved useful for improving nutrient access and optimizing plant architecture, but several fundamental gaps—including the chemical nature of endogenous SLs active in aerial tissues, the largely uncharacterized SL biosynthetic pathways, the functional significance of SL diversity and receptor specificity, and the unknown SL receptors in AMF—continue to hinder targeted decoupling. 

Looking forward, cost-effective production methods and stable formulations are equally critical for both fundamental studies and field deployment. Emerging opportunities, such as SL–microbiome biostimulants and synthetic biology platforms, offer promising avenues for future translation. With continued research and investment, SL-based solutions hold the potential to enhance crop productivity, reduce chemical inputs, and mitigate the impact of parasitic weeds and abiotic stresses in sustainable agriculture.

## Figures and Tables

**Table 1:** Comparison of canonical and non-canonical strigolactones.

Feature	Canonical SLs	Non-Canonical SLs
Chemical structure	Complete ABCD-ring system (tricyclic lactone + butenolide)	Lack one or more of the A, B or C rings; retain D-ring
Key biosynthetic enzymes	MAX1 (CYP711A2/A3), CYP722C, CYP728B, CYP712G, LGS1	MAX1 (CYP711A1), CLAMT, LBO, LLD, CYP706C2/C37
Main biological functions	Rhizosphere signals: induce parasitic weed germination; promote AMF hyphal branching	Endogenous phytohormones: regulate shoot branching; modulate root architecture and mycorrhization
Agricultural significance	Primary target for parasitic weed control	Key for decoupling: can be preserved to maintain tillering while reducing parasitic weed attraction

**Table 2:** Comparison of precision intervention strategies for parasitic weed control using strigolactones.

Strategy	Category	Advantages	Limitations	Field Validation
Biosynthesis editing	Functional decoupling (genetic)	Permanent resistance; can alter SL profile	May affect yield and plant architecture (e.g., *NAB1* mutation in sorghum)	Sorghum (*lgs1* mutant) shows reduced *Striga* damage [63]
Transporter editing	Functional decoupling (genetic)	No yield penalty; preserves intracellular SL functions; prevents reduction in rhizosphere SL exudation	Requires species-specific transporter identification	Sorghum (*SbSLT1/2* KO) and tomato (*SlABCG45* KO) field trials with significantly reduced infestation [22,23]
Receptor-specific mimics	Functional decoupling (chemical)	Pathway-specific activation; minimal off-target effects	Most compounds remain at laboratory stage	SPL7 validated in pot/lab experiments for *Striga*-selective germination [66]; 4BD, AR36, 113D10 at lab stage [67,68,69]
Suicidal germination	Situational decoupling	Easy application; non-genetic; depletes soil seed bank	High synthesis cost; limited soil stability; efficacy varies by location	MP3 and Nijmegen-1 in maize (Kenya) [20], pearl millet and sorghum (Burkina Faso) [21]

**Table 3:** Representative studies on exogenous GR24 application for drought stress mitigation.

Crop Species	SL Compound	Key Phenotypic Effects	Experimental Setting	Reference
Wheat	GR24	Enhanced antioxidant enzyme activities, reduced lipid peroxidation, improved photosynthetic efficiency, and increased grain yield	Pot/greenhouse	[76,77]
Wheat	GR24	Enhanced drought tolerance when combined with ABA and AMF inoculation	Pot/greenhouse	[78]
Wheat	GR24	Maintained photosynthesis and yield	Pot/greenhouse	[79]
Wheat	GR24	Promoted grain filling, increased kernel weight and final yield	Pot/greenhouse	[80]
Maize	AB01	Increased grain yield	Field trial	[24]
Maize	GR24	Enhanced photosynthetic pigments, water use efficiency, cob diameter, cob weight, and seed number per cob/plant	Pot/greenhouse	[81]
Maize	GR24	Improved post-drought recovery	Pot/greenhouse	[82]
Soybean	GR24	Enhanced osmotic balance, ROS scavenging, and root growth	Pot/greenhouse	[83]
Cotton	GR24	Improved photosynthetic efficiency, promoted stomatal closure, activated antioxidant defenses	Pot/greenhouse	[84]
Grapevine	GR24	Mitigated decline in relative water content, protected photosystems	Pot/greenhouse	[85]

## Data Availability

No new data were created or analyzed in this study.
